# ROS-Mediated Inhibition of *S*-nitrosoglutathione Reductase Contributes to the Activation of Anti-oxidative Mechanisms

**DOI:** 10.3389/fpls.2016.01669

**Published:** 2016-11-10

**Authors:** Izabella Kovacs, Christian Holzmeister, Markus Wirtz, Arie Geerlof, Thomas Fröhlich, Gaby Römling, Gitto T. Kuruthukulangarakoola, Eric Linster, Rüdiger Hell, Georg J. Arnold, Jörg Durner, Christian Lindermayr

**Affiliations:** ^1^Institute of Biochemical Plant Pathology, Helmholtz Zentrum München – German Research Center for Environmental HealthNeuherberg, Germany; ^2^Centre for Organismal Studies Heidelberg, Ruprecht-Karls-Universität HeidelbergHeidelberg, Germany; ^3^Institute of Structural Biology, Helmholtz Zentrum München – German Research Center for Environmental HealthNeuherberg, Germany; ^4^Laboratory for Functional Genome Analysis, Gene Center, Ludwig-Maximilians-Universität MünchenMunich, Germany; ^5^Lehrstuhl für Biochemische Pflanzenpathologie, Technische Universität MünchenFreising, Germany

**Keywords:** nitric oxide, *S*-nitrosoglutathione reductase, *S*-nitrosothiols, reactive oxygen species, oxidative stress, hydrogen peroxide, paraquat, *Arabidopsis thaliana*

## Abstract

Nitric oxide (NO) has emerged as a signaling molecule in plants being involved in diverse physiological processes like germination, root growth, stomata closing and response to biotic and abiotic stress. *S*-nitrosoglutathione (GSNO) as a biological NO donor has a very important function in NO signaling since it can transfer its NO moiety to other proteins (*trans*-nitrosylation). Such *trans*-nitrosylation reactions are equilibrium reactions and depend on GSNO level. The breakdown of GSNO and thus the level of *S*-nitrosylated proteins are regulated by GSNO-reductase (GSNOR). In this way, this enzyme controls *S*-nitrosothiol levels and regulates NO signaling. Here we report that *Arabidopsis thaliana* GSNOR activity is reversibly inhibited by H_2_O_2_ *in vitro* and by paraquat-induced oxidative stress *in vivo*. Light scattering analyses of reduced and oxidized recombinant GSNOR demonstrated that GSNOR proteins form dimers under both reducing and oxidizing conditions. Moreover, mass spectrometric analyses revealed that H_2_O_2_-treatment increased the amount of oxidative modifications on Zn^2+^-coordinating Cys47 and Cys177. Inhibition of GSNOR results in enhanced levels of *S*-nitrosothiols followed by accumulation of glutathione. Moreover, transcript levels of redox-regulated genes and activities of glutathione-dependent enzymes are increased in *gsnor-ko* plants, which may contribute to the enhanced resistance against oxidative stress. In sum, our results demonstrate that reactive oxygen species (ROS)-dependent inhibition of GSNOR is playing an important role in activation of anti-oxidative mechanisms to damping oxidative damage and imply a direct crosstalk between ROS- and NO-signaling.

## Introduction

Plants are continuously facing to the changing environment that affects plant growth and productivity. To deal with multiple stress conditions, plants have developed adaptive responses. Nitric oxide (NO) as a signaling molecule plays a crucial role in these responses acting alone or together with reactive oxygen species (ROS) to regulate hormonal signaling pathways, gene expression changes or protein activities. The regulatory role of NO has been demonstrated in response to abiotic and biotic stresses as well as in plant developmentally processes throughout the entire plant life (Corpas et al., 2011; Yu et al., 2014; Simontacchi et al., 2015). NO can influence protein activity, translocation and protein function by posttranslational modifications. The predominant way of NO action is the reversible *S*-nitrosylation, a covalent attachment of NO to cysteine thiols. Further modifications are the nitrosylation of metal center of metalloproteins and the irreversible nitration of protein tyrosine residues (Astier and Lindermayr, 2012; Kovacs and Lindermayr, 2013). As a free radical, NO has a very short lifetime that restrict their effect to the local microenvironment. However, *S*-nitrosylated glutathione (*S*-nitrosoglutathione GSNO) is a quite stable NO reservoir and NO transport form. GSNO can *trans*-nitrosylate proteins regulating their activity/function (Lindermayr et al., 2010; Espunya et al., 2012; Corpas et al., 2013; Frungillo et al., 2014). GSNO level is regulated either by its production or by an enzymatic turnover mechanism catalyzed by GSNO reductase (GSNOR). Mutations in *GSNOR* gene have been shown to cause pleiotropic plant growth defects, impaired plant disease responses, heat sensitivity, and resistance to cell death (Feechan et al., 2005; Rusterucci et al., 2007; Lee et al., 2008; Kwon et al., 2012; Xu et al., 2013). The *gsnor-ko* plants contain elevated amount of *S*-nitrosothiols (SNO) and nitroso species indicating that GSNOR activity controls the level of both GSNO and indirectly protein-SNOs (Liu et al., 2001; Feechan et al., 2005; Lee et al., 2008). GSNOR, originally identified in plants and other organisms as a glutathione-dependent formaldehyde dehydrogenase (GS-FDH), belongs to the class III alcohol dehydrogenase family (EC 1.1.1.1) (Martinez et al., 1996). The crystal structure of GS-FDH from mammals, yeast and plants revealed that the enzyme is a homodimer coordinating two zinc atoms per subunit (Sanghani et al., 2002; Kubienova et al., 2013). Few years later, evidence was provided that GS-FDH is involved also in the *S*-nitrosothiol metabolism (Liu et al., 2001) and GSNO degrading activity was described for *Arabidopsis* GS-FDH (Sakamoto et al., 2002). GSNOR is a highly conserved enzyme in mammals, yeast and plants and is essential to protect cells under nitrosative stress (Liu et al., 2001; Corpas et al., 2011).

Reactive oxygen species as oxidants and signaling molecules have a fundamental influence in almost all biological processes (Apel and Hirt, 2004). The regulated production of ROS due to biotic and abiotic stimuli is necessary to activate downstream responses (Shaikhali et al., 2012; Foyer and Noctor, 2013; Dietz, 2014). However, the excessive accumulation of ROS can lead to detrimental consequences; therefore, precise regulation of ROS level is highly important. Next to the enzymatic decomposition of ROS by catalase, ascorbate peroxidase (APX) or other enzymes in the glutathione-ascorbate cycle, the non-enzymatic way by low molecular weight antioxidants, like glutathione (GSH) and ascorbate has crucial role to balance cellular redox changes (Foyer and Noctor, 2013). ROS can modify cysteine thiols and methionine residues of redox sensitive target proteins resulting in oxidative posttranslational modifications or irreversible oxidations of proteins (Konig et al., 2012; Waszczak et al., 2015). It has been shown that these oxidative modifications affect enzyme or metal-binding activity of important signaling proteins, like protein phosphatases and mitogen-activated protein kinases (Gupta and Luan, 2003; Jammes et al., 2009; Waszczak et al., 2014) or transcription factors (Dietz, 2014).

H_2_O_2_ and NO are commonly produced during various stress conditions suggesting a strong interplay between both signaling molecules. NO accumulation induced by *Verticillium dahlia* toxin depends on prior H_2_O_2_ production (Yao et al., 2012). Further evidences supported cross-talks of ROS and NO in cryptogein-induced defense response of tobacco cells (Kulik et al., 2014) and also in systemic acquired resistance in *Arabidopsis* (Wang et al., 2014). H_2_O_2_-induced NO production mediates abscisic acid-induced activation of a mitogen-activated protein kinase cascade (Zhang et al., 2007) and contributes to hydrogen-promoted stomatal closure (Xie et al., 2014). Despite the evidences of the crosstalk of ROS and NO signaling, there are still gaps in that regard how they control each other level and what is the consequence of their interactions.

Therefore, the focus of this study was to investigate a direct impact of ROS on GSNOR protein and thereby on cellular NO metabolism. We show that GSNOR activity is inhibited by paraquat-induced oxidative burst in wild type *Arabidopsis* seedlings accompanied by an increased cellular *S*-nitrosothiol and nitrite level. Furthermore, *gsnor* plants accumulate GSH, which acts as redox buffer to scavenge RNS. Transcripts encoding for redox-related proteins and activities of GSH-dependent enzymes were increased. Furthermore, we measured GSNOR activity under oxidizing conditions and analyzed cysteine residues by LC-MS/MS for potential oxidative modifications. We demonstrated that oxidative conditions inhibited GSNOR activity *in vitro* and this inhibition correlated with Zn^2+^ release of GSNOR. In sum, ROS-dependent regulation of GSNOR contributes to fine-tuning of NO/SNO levels, which can act directly as a ROS scavenger and/or activate antioxidant mechanisms in response to oxidative stress.

## Materials and Methods

### Plant Material and Growth Conditions

*Arabidopsis thaliana* (L.) Heynh (ecotype Columbia-0 and Wassilewskija) wild type seeds and knock-out mutants of the *GSNOR* gene (At5g43940) were obtained from GABI-kat (GABI-Kat 315D11, background Columbia-0) and FLAG T-DNA collections (Versailles Genomic Resource Centre; FLAG_298F11, background Wassilewskija). After vernalisation for 2 days (4°C in dark), plants were cultivated for 4 weeks in a climate chamber at 60% relative humidity under long-day condition (16 h light/8 h dark cycle, 20°C day/18°C night regime, 100 μmol m^-2^ s^-1^ photon flux density). For seed germination analyses, *Arabidopsis* seeds were surface sterilized and grown on half strength MS medium containing 1% sucrose in a climate chamber under long-day condition.

### Paraquat Treatment

Sterile seeds were germinated and grown on half strength MS plates containing different paraquat (methyl viologen, Sigma–Aldrich, Steinheim, Germany) concentrations (0.25–10 μM) for 1 to 2 weeks. To test tyrosine nitration, Western blot was made using anti-nitrotyrosine antibody (Merck Millipore, Darmstadt, Germany) as described (Holzmeister et al., 2015). For spray application, 1, 10, and 50 μM paraquat or water (control) was sprayed onto the leaf surface of 4-week-old plants. Leaves were collected after 1-day of treatment, frozen and kept at -80°C until use. For Western-blot analysis of total protein extract made from paraquat-treated leaves, polyclonal antibody against *Arabidopsis* GSNOR (Agrisera, Sweden) was used.

### NO Fumigation

Four-week-old plants were placed in an incubator and fumigated with 80 ppm gaseous NO or with synthetic air without NO for 20 min. The experimental setup consisted of controlled-environment cabinets as well as equipment to adjust and control gaseous NO treatment. NO concentration was monitored with a Chemiluminescence Nitrogen Oxides Analyzer AC32M (Ansyco, Karlsruhe, Germany). After fumigation, the plants were placed in the growth chamber until sample collection.

### Cloning of AtGSNOR and Cysteine Mutants GSNOR^C47S^, GSNOR^C177S^, GSNOR^C271S^

Total RNA isolated from wild type *Arabidopsis* leaves was used to produce cDNAs by SuperScript II Reverse transcriptase (Invitrogen, Carlsbad, CA, USA). For amplification of coding sequence of AtGSNOR for gateway cloning, the first PCR reaction was made using gene-specific primers (ADH2-ATG-for: 5′-ATGGCGACTCAAGGTCAG-3′; ADH2-TGA-rev: 5′-TCATTTGCTGGTATCGAGGAC-3′). Afterward, the second PCR reaction was performed to introduce recombination sequences (att) at the 5′- and 3′-end using the following primers (ADH2-GW-forward: 5′-GGGGACAAGTTTGTACAAAAAAGCAGGCTTCATGGCGACTCAAGGTC-3′; ADH2-GW-reverse: 5′- GGGGACCACTTTGTACAAGAAAGCTGGGTCTCATTTGCTGGTATCGAG-3′). The resulting PCR products were cloned into pDONR221 vector (Invitrogen, Carlsbad, CA, USA) by recombination reaction using BP Clonase enzyme mixture according to the instructions of the manufacturer. After sequencing, the correct clone was transferred into the expression vector pDEST17 by recombination using LP Clonase enzyme mixture (Invitrogen, Carlsbad, CA, USA).

#### Site-Directed Mutagenesis

The modification of single nucleotide residues was performed as previously described (Lindermayr et al., 2003). Briefly, for mutation, a complementary pair of oligonucleotides was synthesized harboring the desired alterations. For amplification, 20 ng plasmid DNA was used in a total volume of 15 μl, including 1 μM each primer, 200 μM dNTPs, and 1 U of PfuTurbo DNA polymerase. After denaturation (2 min at 94°C) 18 cycles were conducted, consisting of 45 s at 94°C, 30 s at 55°C, and 15 min at 72°C, followed by a final extension step at 72°C for 10 min. Subsequently, the parental and hemi-parental DNA was digested with DpnI and the amplified plasmids were transformed into *Escherichia coli* DH5α. The mutation was verified by sequencing. The next primers were used to make GSNOR^C47S^ forward: CTACACTGCTCTTAGTCACACCGACGCTTAC and reverse: GTAAGCGTCGGTGTGACTAAGAGCAGTGTAG; for GSNOR^C177S^ forward: GTTTGCCTTCTTGGAAGTGGTGTTCCCACTG and reverse: CAGTGGGAACACCACTTCCAAGAAGGCAAAC; for GSNOR^C271S^ forward: GACTACAGCTTTGAGAGCATCGGGAATGTCTC and reverse: GAGACATTCCCGATGCTCTCAAAGCTGTAGTC.

### Purification of Recombinant GSNOR and Cysteine Mutants

For expression of the recombinant N-terminal His_6_ fusion proteins the wild type pDEST17-GSNOR and the cysteine mutants GSNOR^C47S^, GSNOR^C177S^, GSNOR^C271S^ were transformed into the *E. coli* strain BL21 DE3 pLysS., The LB cultures at *A*_600_ ∼0.6 were induced with 0.1 mM isopropyl-β-D-thiogalactopyranoside and further incubate for 4 h at 28°C. After induction the bacterial cells were harvested by centrifugation and stored frozen. For protein isolation the cells were resuspended in a lysis buffer [25 mM Tris-HCl, pH 8.0, 1 mM EDTA, 0.5% Triton X-100, 20% (v/v) glycerol, 1 mM β-mercaptoethanol] and disrupted by sonication. Cellular debris was removed by centrifugation and the soluble fraction was purified by affinity chromatography using Ni-NTA agarose (Qiagen, Hilden, Germany). Adsorbed proteins were eluted from the matrix with elution buffer containing 25 mM Tris-HCl pH 8.0, 250 mM imidazole, 10 mM DTT, 20% (v/v) glycerol. The eluates were aliquoted, frozen in liquid nitrogen and stored at -80 °C until analysis. The purity was checked by SDS-PAGE and the protein concentration was measured by Bradford assay (Bio-Rad). Before the further use the elutions were desalted by Zeba-Spin column (Thermo Scientific, Rockford, IL, USA).

### Static Light-Scattering Analysis

Static light scattering (SLS) experiments on recombinant GSNOR were performed at 30°C using a Viscotek TDA 305 triple array detector (Malvern Instruments) downstream to an Äkta Purifier (GE Healthcare) equipped with an analytical size exclusion column (Superdex 200 10/300 GL, GE Healthcare) at 4°C. GSNOR protein was purified under reducing condition, followed by Äkta purification, than 200 μg GSNOR was oxidized by 1 mM H_2_O_2_. The reduced and oxidized GSNOR samples were run in 50mM Tris-HCl pH 8.0, 200 mM NaCl, with or without 10 mM DTT, respectively, at a flow rate of 0.5 ml/min. The molecular masses of the samples were calculated from the refractive index and right-angle light-scattering signals using Omnisec (Malvern Instruments). The SLS detector was calibrated with a 4 mg/ml BSA solution with 66.4 kDa for the BSA monomer and a dn/dc value of 0.185 ml/g for all protein samples.

### Enzyme Activity Assays

Purified GSNOR protein was re-buffered using Zeba Spin column equilibrated with 20 mM Tris-HCl pH 8.0 buffer. GSNOR activity was determined by measuring the reaction rate of NADH usage at 340 nm in Ultrospec 3100 pro (Amersham Biosciences) spectrophotometer. The reaction buffer contained 20 mM Tris-HCl pH 8.0, 0.5 mM EDTA, 0.2 mM NADH. Serial dilutions (50–2000-fold) of recombinant His-tagged GSNOR proteins (wild type and cysteine mutants) were prepared and the reaction was started to add GSNO (Enzo Life Sciences) at a final concentration of up to 0.5 mM. Water was used instead of GSNO in the reference sample. The reaction was monitored for 5 min and the linear rate was corrected with a reference rate without GSNO. There was no detectable NADH oxidation without enzyme. Specific activity was calculated using a molar extinction coefficient for NADH 6.22 mM^-1^cm^-1^. Effect of H_2_O_2_, PN or NEM on GSNOR activity was analyzed by incubation of recombinant GSNOR with these compounds for 20 min in 20 mM Tris-HCl pH 8.0. Excess H_2_O_2_, PN or NEM was removed by gel filtration using Zeba desalting columns (Thermo Fisher Scientific, Waltham, MA, USA). Zeba Spin columns were equilibrated with 20 mM Tris-HCl pH 8.0. The eluates were used to determine GSNOR activity. To analyze reversibility of H_2_O_2_-dependent inhibition of GSNOR, excess H_2_O_2_ was removed (Zeba Spin, 20 mM Tris-HCl pH 8.0) and inhibited GSNOR was divided into two fractions. One fraction was treated with water, the other one was treated with 10 mM DTT for 10 min at room temperature. Before measuring the activity, both samples were desalted using Zeba Spin columns equilibrated with 20 mM Tris-HCl pH 8.0. To analyze the effect of H_2_O_2_ in presence of excess Zn^2+^, GSNOR was treated with 0.5 mM H_2_O_2_ in presence of 0.5 μM ZnSO_4_ for 20 min. Excess H_2_O_2_ and ZnSO_4_ was remove using Zeba Spin columns (20 mM Tris-HCl pH 8.0) and GSNOR activity was determined.

To measure GSNOR activity from plant tissue, total soluble proteins were extracted from treated seedlings or leaves in buffer of 0.1 M Tris–HCl pH 7.5, 0.1 mM EDTA, 0.2% TritonX-100, 10% glycerol. The homogenate was centrifuged twice at 14.000 *g* for 20 min at 4°C and total protein concentration of the supernatant was measured according to Bradford using BSA as a standard. GSNOR activity was determined by incubating 100 μg of protein extract in 1 ml reaction buffer as described above.

Glutathione reductase activity assay is based on the NADPH-dependent reduction of GSSG to GSH. GR activity was measured by the rate of NADPH oxidation at 340 nm. Proteins from 2-week-old seedlings were extracted with extraction buffer (50 mM potassium phosphate pH 7.8, 0.1 mM EDTA, 0.5% Triton X-100, 0.5% PVP-40). 50 μg total protein was incubated in 1 ml GR reaction buffer (100 mM potassium phosphate pH 7.8, 1 mM EDTA, 0.2 mM NADPH). The reaction was started by addition of 100 μl of 5 mM GSSG and was monitored at 340 nm for 5 min. The linear rate of reaction was corrected with a reference rate without GSSG (molar extinction coefficient for NADPH 6.22 mM^-1^cm^-1^).

Glutathione-*S*-Transferase (GST) activity was measured by spectrophotometrically using the artificial substrate 1-chloro-2,4-dinitrobenzene (CDNB) for GSH attachment. 50 μg of total protein was incubated in 1 ml GST reaction buffer (100 mM potassium phosphate pH 7.8, 1 mM CDNB in 80% ethanol) and the reaction was started by adding 100 μl of 10 mM GSH. The production of GSH-CDNB conjugate was monitored at 340 nm [molar extinction coefficient (𝜀) = 9600 M^-1^ cm^-1^].

### Zn^2+^ Release Assay

Free Zn^2+^ ions were detected by a metallochromic indicator 4-(2-pyridylazo)-resorcinol (PAR), which binds Zn^2+^ in a 2:1 complex and turn its color from yellow to orange with strong absorbance at 490 nm (Crow et al., 1997). Recombinant GSNOR (50–100 μg) was oxidized by increasing molar excess (100–3000 to protein) of H_2_O_2_ for 1 h in 50 mM Tris-HCl pH7.2 and mix with 100 μM PAR. The absorbance of PAR_2_-Zn complex was measured at 490 nm and the Zn content was calculated using ZnCl_2_ standard curve.

### Mass Spectrometric Analyses of GSNOR

Twenty microliter of H_2_O_2_-treated (molar ratio of H_2_O_2_ to protein was 100:1 or 1000:1) or water-treated (as control) recombinant GSNOR (corresponding to approximately 8 μg of protein) was incubated with 20 μl of non-reducing SDS-gel loading buffer (20% glycerol, 4% SDS, 0.125 M Tris-HCl pH 6.8) containing 55 mM 2-iodoacetamide (IAA). To remove the excess of IAA completely, samples were separated under non-reducing conditions on SERVAGel TG PRiME 4-12% (SERVA, Heidelberg, Germany). Then the gel was stained with Coomassie overnight and after de-staining the bands were excised and transferred into 1.5 mL reaction tubes. Reduction of cysteine residues was performed for 30 min at 55°C using 45 mM DTT in 50 mM NH_4_HCO_3_. After removal of DTT solution, blocking was performed using 50 mM *S*-methyl-methanethiosulfonate (MMTS) for 30 min, then the gel slices were washed three times using 50 mM NH_4_HCO_3_. The in-gel digestion was performed overnight using 500 ng bovine chymotrypsin (Roche Diagnostics, Mannheim, Germany) or 70 ng trypsin (Promega, Fitchburg, WI, USA). Peptides were separated on NanoLC Ultra chromatography system (Eksigent, Redwood City, CA, USA) coupled to an LTQ Orbitrap XL mass analyzer (Thermo Fisher Scientific, San Jose, CA, USA). Mobile phase A was 0.1% formic acid and mobile phase B was 84% acetonitrile/0.1% formic acid. For separation, a reversed phase nano-column (Reprosil-Pur C18 AQ, 2.4 μm; 150 mm × 75 μm, Dr. Maisch, Ammerbuch-Entringen, Germany) at a flow rate of 280 nl/min was used. The separation method consisted of two linear gradients (1–30% B in 120 min and 30–60% B in 10 min). Mass spectra were acquired in cycles of one MS Orbitrap scan, followed by five data dependent ion trap MS/MS scans (CID, collision energy of 35%). MS spectra were searched using MASCOT 2.4 (Matrix Science, London, UK) using the *A. thaliana* subset of the SwissProt Database and the following parameters: (a) Variable modifications: Dioxidation (C), Trioxidation (C), Methylthio (C), Carbamidomethyl (C), Oxidation (M); (b) Enzyme: none, (c) Peptide charge: 1+, 2+, and 3+; (d) Peptide tol. ± : 10 ppm; (e) MS/MS tol. ± : 0.8 Da. MASCOT DAT files were imported into the Scaffold software package (Proteome Software Inc., Portland, OR, USA) and filtered for hits with a confidence of 99% at the protein level and 95% for individual peptides.

### Microarray Analysis

Total RNA from 4 to 5-week old rosette leaves of Wassilewskija *Arabidopsis* WT and *gsnor* was isolated using RNeasy Plant Mini Kit (Qiagen, Germany) according to the manufacturer’s instructions. Quality checking and quantification of RNA isolates were carried out using Agilent RNA 6000 Nano kit on Agilent 2100 BioAnalyzer. Microarray analysis was performed on Agilent platform using the technique “One color Microarray-based Gene Expression Analysis” according to the protocol described in the Agilent manual. The raw expression data of three biological replicates per genotype was analyzed using GeneSpring GX software tool. Statistical analysis were carried out to identify the differentially expressed genes (*p* < 0.05) between the two genotype using One Way Anova analysis with the Benjamini-Hochberg multiple test correction (FDR) and SNP *Post hoc* test. From the gene list, those ones regulated at least by twofold differences were selected for downstream analysis. Microarray data are available in the ArrayExpress database^1^ under accession number E-MTAB-4756.

### Determination of Glutathione

The amount of the reduced and oxidized glutathione was determined in 2-week-old seedlings using the GR-based recycling assay described previously (Queval and Noctor, 2007). Furthermore, total glutathione, cysteine and γ-glutamyl-cysteine content were measured by reverse-phase HPLC after NO fumigation experiment. Briefly, 200 mg leaf material was extracted in 0.1 M HCl. Subsequently, all low molecular weight thiols were reduced by addition of DTT and then derivatized with 10 mM monobromobimane as described previously (Wirtz et al., 2004). Samples were analyzed by reverse-phase HPLC and fluorescence excitation at 380 nm.

### Determination of Ascorbate

The amount of the reduced and total ascorbate was determined as described by Queval and Noctor (2007). Four weeks old *Arabidopsis* plants were sprayed with 0, 1, 10, or 50 μM of paraquat, harvested and stored in -80°C until use. The amount of reduced ascorbate (ASC) was measured at 265 nm in a Tecan plate reader before and after incubation with ASC oxidase. ASC oxidase converts the reduced ASC to the non-absorbing oxidized form. For determination of total ASC, the oxidized ASC was first reduced to ASC by adding 1 mM DTT for 30 min, then total ASC was measured as above. ASC standard was used to calculate the amount of ASC.

### *In situ* Staining of Diaminobenzidine (DAB) and Nitroblue Tetrazolium (NBT)

For detection of H_2_O_2_, paraquat or water treated *Arabidopsis* seedlings were vacuum infiltrated with 0.1% 3,3′-Diaminobenzidine (DAB) in 10 mM MES pH 6.5 solution, washed three times with water and incubated for 45 min at RT in light. After staining, plants were destained with 90% ethanol at 60°C. The brown precipitate shows the presence of H_2_O_2_ in the cell and tissue.

*Arabidopsis* plants were vacuum infiltrated with nitroblue tetrazolium (NBT) [50 mM potassium phosphate/pH 6,4; 10 mM NaN_3_; 0,1% (w/v) NBT] solution, incubated for 45 min in dark and washed three times with water. Afterward, plants were de-stained with 90% ethanol at 60°C.

### Determination of Nitrosothiols and Nitrite

Total nitrite, nitrate, and nitrosothiol content were measured using a Sievers 280i nitric oxide analyser (GE Analytical Instruments, Boulder, CO, USA). Proteins were extracted from rosettes using extraction buffer (137 mM NaCl, 0,027 mM KCl, 0,081 mM Na_2_HPO_4_.2H_2_O, 0,018 mM NaH_2_PO_4_). Leaf protein extract was injected into the purging vessel containing 3.5 ml of acidified KI/I_3_^-^ solution (reducing agent) at 30°C. The recorded mV signals were plotted against a calibration curve produced using known concentrations of sodium nitrite solution to quantify the nitrite level. To estimate the *S*-nitrosothiol content (RSNO), the above protocol was repeated by pre-treating the leaf protein extract with 20 mM sulphanilamide (in 1 M HCl) at the ratio of 9:1. For nitrate quantification, the reducing agent was replaced with vanadium chloride at 95°C. The recorded mV signals were plotted against a calibration curve produced using known concentrations of sodium nitrate solution to quantify the nitrate levels.

### Statistical Analysis

For multiple comparisons, analysis of variance was performed by Anova (one way or two way) followed by Holm-Sidak test. For pairwise comparison, Student’s *t*-test was used. The level of significance is indicated in each figure.

## Results

### Paraquat-Induced Inhibition of GSNOR Activity

**FIGURE 1:**
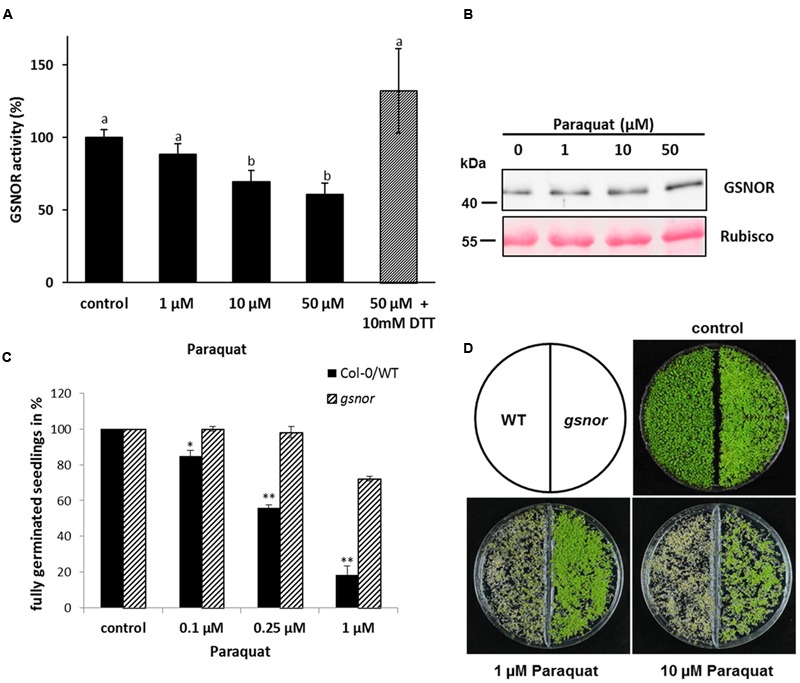
**Inhibition of GSNO-reductase (GSNOR) activity *in vivo* by paraquat and tolerance of *gsnor* mutants to oxidative stress. (A)** Measurement of GSNOR activity from crude leaf extracts of 4-week-old *Arabidopsis* plants exposed to different paraquat concentrations for 24 h. For restoring enzyme activity, 10 mM DTT was added to the extract before the measurement (gray bar). Values are expressed as percentage of water treated control plants (specific activity varies 49.2–91.1 nmol NADH min^-1^ mg^-1^) and represent the mean ± SD calculated from three biological replicates. Different letters indicate significant differences, *p* < 0.05, Anova. **(B)** Western blot of paraquat-treated plant extracts using GSNOR-specific antibody. Ponceau staining of Rubisco protein represents the equal loading. **(C)** Germination rates of 1-week-old Col-0 WT and *gsnor* mutant growing on 0.1, 0.25, and 1 μM paraquat-containing media. The germination rate was calculated by counting fully germinated seedlings with open cotyledons and is presented in percentage of control (without paraquat). ^∗^*p* < 0.05, ^∗∗^*p* < 0.005 indicate significant differences between WT and *gsnor*. **(D)** Representative pictures of WT and *gsnor* seedlings germinated on paraquat-containing media. Control is without paraquat.

*S*-nitrosoglutathione-reductase controls intracellular levels of SNOs and thereby this enzyme is important for NO homeostasis. Paraquat (1,1′-dimethyl-4,4′-bipyridinium dichloride) is an herbicide, which induces the accumulation of ROS, such as superoxide, H_2_O_2_ and other deleterious oxygen radicals (Dodge, 1971; Babbs et al., 1989) and is a good tool to investigate the effect of ROS on GSNOR activity *in vivo*. Four week old *Arabidopsis* plants (Col-0) were sprayed with this herbicide. Paraquat treatment for 24 h significantly decreased GSNOR activity in a dose-dependent manner (**Figure 1A**). Application of 50 μM paraquat resulted in 40% enzyme inhibition, which could be restored with 10 mM of the reducing agent 1,4-dithiothreitol (DTT) suggesting that oxidative modification(s) are responsible for inhibition of GSNOR activity. Moreover, immunoblot analysis using GSNOR-specific antibody demonstrated only a slight change of GSNOR protein amount during paraquat treatment (**Figure 1B**). The accumulation after treatment with 50 μM paraquat is around 1.3-fold in comparison to the control sample and might partly compensate for the paraquat-induced inhibition of GSNOR.

To study the physiological function of paraquat/ROS-induced GSNOR inhibition, plants lacking GSNOR function were analyzed for their response to paraquat treatment. Interestingly, Chen et al. (2009) previously observed that GSNOR plays a role in regulating paraquat-induced cell death in plant cells through modulating intracellular NO level. We used two T-DNA insertion alleles for *GSNOR* (background Col-0 and Wassilewskija, named *gsnor* and WS/*gsnor*, respectively) to test their germination and growth in presence of paraquat. Seeds of WT and *gsnor* plants were cultivated on MS media containing 0–1 μM paraquat and the germination rate was determined by counting fully germinated seedlings with two open cotyledons. The germination rate of WT plants was strongly reduced to 20% in presence of 1 μM paraquat (**Figure 1C**). In contrast, the germination rate of *gsnor* plants was significantly higher (72%) at this paraquat concentration (**Figure 1C**). Similar results could be observed using the T-DNA insertion line in Wassilewskija background (WS/*gsnor*; Supplementary Figure S1A). Paraquat induced cell death phenotype of WT seedlings was obvious using higher paraquat concentration (1 and 10 μM) by the yellowish-brown colored cotyledons and restricted growth (**Figure 1D**). Interestingly, *gsnor* mutant showed an enhanced tolerance even in the presence of 10 μM paraquat demonstrated by green viable seedlings.

Paraquat-induced O_2_^-^ can react with NO resulting in ONOO^-^ production, which can oxidize cysteine residues or nitrate tyrosine residues of proteins. Protein tyrosine nitration is a marker for pathological processes in cell death. WT seedlings germinated on 0.5 μM paraquat showed stronger tyrosine nitration than *gsnor* seedlings (Supplementary Figure S1B) suggesting a weaker cell death phenotype for *gsnor* mutant, which correlates with the observed visible effect of more green seedlings (**Figure 1D**).

### Paraquat-Induced Changes in NO Metabolism

**FIGURE 2:**
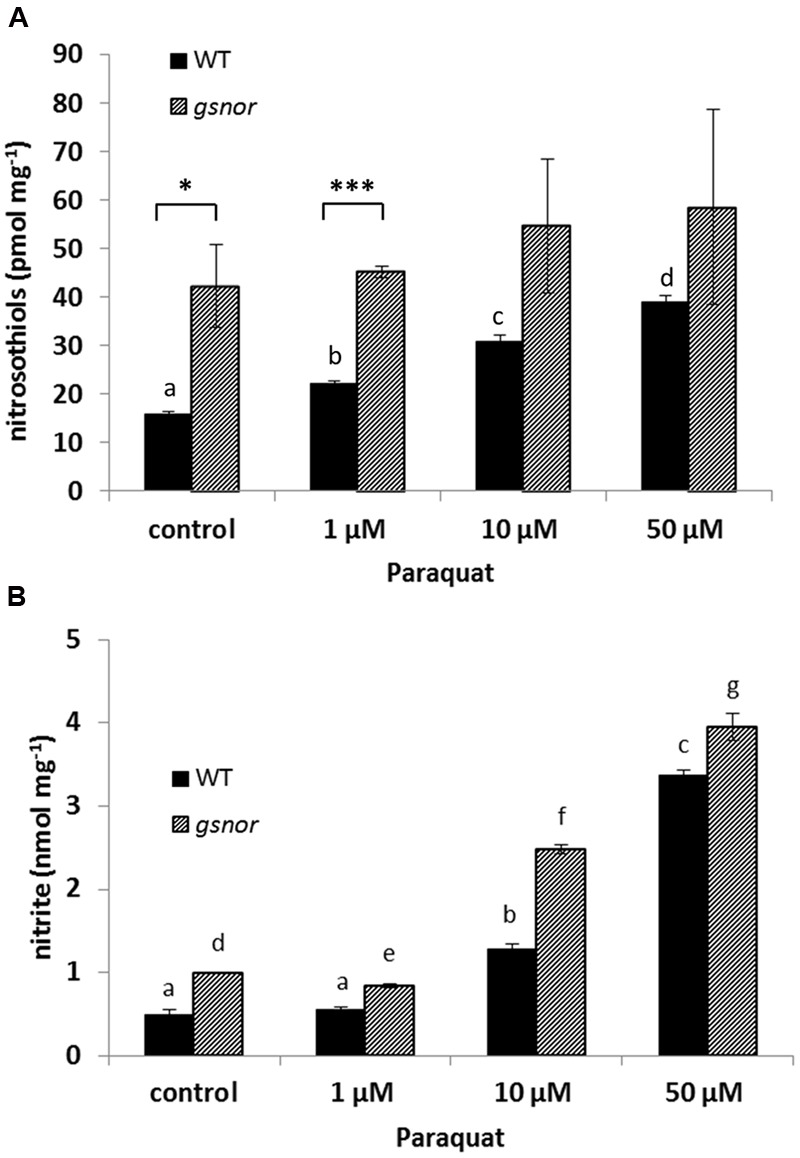
**Paraquat-induced changes in NO metabolism.** Crude leaf extracts from 4-week-old WT (black bar) and *gsnor* (gray bar) plants exposed to different paraquat concentrations (1, 10, and 50 μM) for 24 h were used for determination of nitrosothiol **(A)** and nitrite **(B)** contents. Values were normalized against total protein content and represent the mean ± SD calculated from three replicates. The different letters indicate significant differences among the samples (*p* < 0.05, Anova) and ^∗^*p* < 0.05, ^∗∗∗^*p* < 0.001 represent significant differences between WT and *gsnor* (a).

Since GSNOR activity is important for SNO homeostasis, intracellular SNO levels were analyzed after paraquat treatment. WT and *gsnor* plants were sprayed with 1, 10, and 50 μM paraquat and total cellular SNO content was determined. SNO levels increased in paraquat-treated WT plants from 15 pmol mg^-1^ up to 40 pmol mg^-1^ (50 μM paraquat) (**Figure 2A**). The *gsnor* mutant has already elevated SNO content under control condition (around 40 pmol mg^-1^) correlating to the loss of GSNOR function. Paraquat application did not result in significant further accumulation of SNOs in *gsnor* plants (**Figure 2A**). In the living cell NO can be oxidized to give nitrite, which would indicate freshly produced NO. Therefore, we measured nitrite content from WT and *gsnor* plants treated with paraquat. The nitrite level increased in both plant lines in a similar degree in the presence of increasing herbicide concentrations (**Figure 2B**); however, the nitrite content was significantly higher over the treatment in *gsnor* plants.

### *gsnor* Plants Have Higher GSH-Dependent Antioxidant Capacity

**FIGURE 3:**
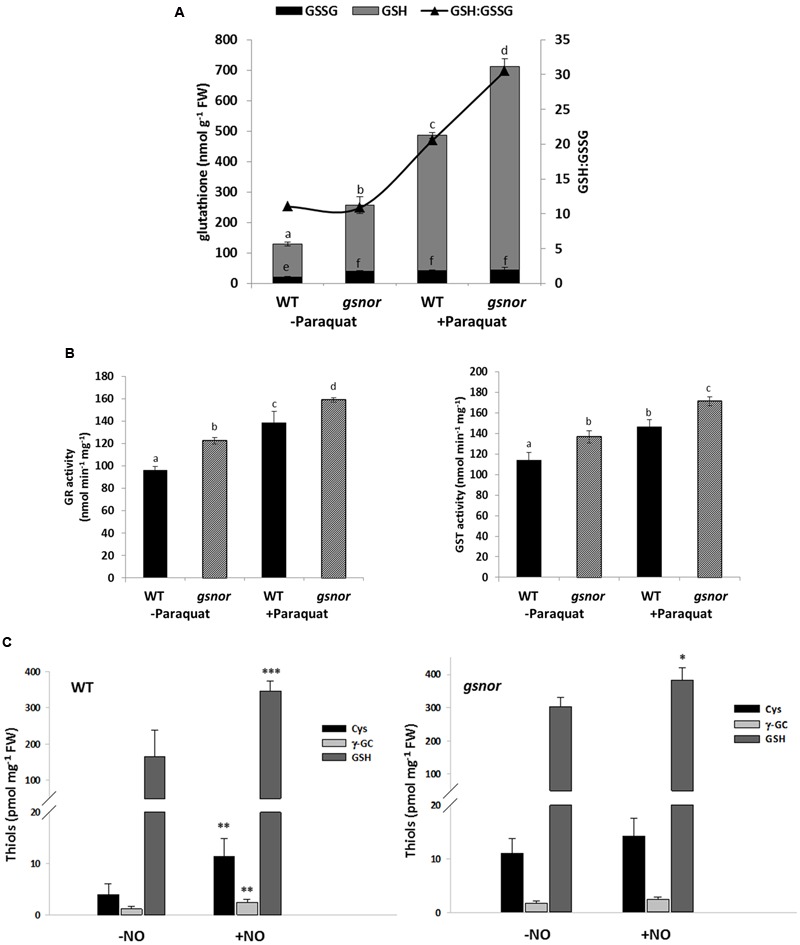
***gsnor* plants has an elevated glutathione-dependent antioxidant capacity to cope with oxidative stress. (A)** Determination of reduced (GSH) and oxidized (GSSG) glutathione from crude extracts of 14-day-old WT and *gsnor* plants were grown on MS media with and without 0,5 μM paraquat. The values were normalized against fresh weight (FW). The ratio of GSH to GSSG (GSH:GSSG, black triangle) is presented on the right axis. **(B)** Enzyme activities for glutathione reductase (GR, left panel) and glutathione-*S*-transferase (GST, right panel) were determined from the same treatments as in **(A)**. The values of enzyme activity represent the mean ± SD calculated from three biological replicates. The different letters indicate significant differences among the samples (*p* < 0.05, Anova). **(C)** Analysis of thiol-containing intermediates of the GSH biosynthesis pathway by HPLC. Four week-old WT plants (left panel) and *gsnor* mutants (right panel) were fumigated with and without 80 ppm NO gas for 20 min. After 1 h of regeneration rosettes were harvested for determination of cysteine (Cys), γ-glutamylcysteine (γ-GC), and total glutathione (GSH) content. Values were normalized against total fresh weight and represent the mean ± SD calculated from three to five samples of each line. ^∗^*p* < 0.05, ^∗∗^*p* < 0.005, ^∗∗∗^*p* < 0.001 represent significant differences between control (-NO) and NO-treated samples (*t*-test).

Resistance against oxidative stress is often related to an enhanced cellular antioxidant capacity. Therefore, the amount of reduced and oxidized glutathione (GSH and GSSG, respectively) was measured in WT and *gsnor* seedlings germinated on media with and without 0.5 μM paraquat for 14 days. GSH level of untreated *gsnor* plants was about twofold higher than of WT plants and the content increased upon paraquat treatment about four and threefold in WT and *gsnor* plants, respectively (**Figure 3A**). About 10% of the glutathione pool was oxidized under control conditions in both plant lines demonstrating that the redox status is the same in WT and *gsnor* plants (GSH:GSSG ratio, **Figure 3A**). Germination on paraquat-containing media for 14 days resulted in increased total glutathione content in both plant lines; however, the glutathione pool was more reducing in the *gsnor* mutant than in WT (GSH:GSSG ratio around 30 and 20, respectively, **Figure 3A**). This result indicates that *gsnor* may cope better with a long-term paraquat treatment than WT plants to keep cellular redox condition more reducing.

In correlation with the increased level of glutathione, we measured around 20% higher activity for glutathione reductase (GR) enzyme in untreated *gsnor* plants in comparison to WT plants (**Figure 3B**). GR reduces GSSG to GSH in a NADPH-dependent manner. In both lines, GR activity increased by around 30% after paraquat treatment. The glutathione *S*-transferase (GST) activity was also higher (with 15%) in untreated *gsnor* plants than in WT and the GST activity increased in both plant lines after paraquat-treatment by around 10% (**Figure 3B**). Ascorbate is another important cellular reductant inter-connected to the anti-oxidative response in chloroplast. However, although the levels of reduced ascorbate were higher in *gsnor* plants after paraquat-treatment, the differences were significant only at high paraquat concentrations (Supplementary Figure S2). All these results suggest that the enhanced GSH levels and enhanced activities of GSH-dependent enzymes contribute to the paraquat tolerant phenotype of *gsnor* plants.

Taken into consideration that loss of GSNOR function results in elevated SNO/NO and GSH levels, which are both important for resistance against paraquat-induced oxidative stress, we analyzed the interplay between both components. Four-week-old WT and *gsnor* plants were fumigated with 80 ppm NO gas for 20 min to mimic NO burst and levels of low molecular weight thiols of the GSH biosynthesis pathway such as cysteine, γ-glutamylcysteine and total glutathione were determined. NO fumigation resulted in an increase of these compounds in both plant lines (**Figure 3C**). In addition, untreated *gsnor* plants had around twofold higher levels of cysteine and glutathione than WT plants, indicating a connection between SNO/NO and the GSH biosynthesis pathway.

### Genes Involved in Antioxidant Mechanisms Are Upregulated in *gsnor* Mutant

To further demonstrate the presence of a pre-induced antioxidant system in *gsnor* plants, a microarray analysis was performed of 4-week-old rosettes of *gsnor* and WT plants. Out of 2159 genes, which were differentially regulated, 1407 genes were significantly upregulated and 752 genes were significantly downregulated by at least twofold in *gsnor* mutant (Supplementary Table S1) (ArrayExpress accession number E-MTAB-4756). Gene enrichment analysis of the regulated genes using VirtualPlant 1.3 platform (Katari et al., 2010) revealed that the most significantly enriched functional categories of the upregulated genes were the catalytic-, hydrolase-, oxidoreductase-, and glutathione transferase-activities (Supplementary Table S2). Among these functional categories, we focused on genes related to processes metabolizing H_2_O_2_. Within the upregulated genes, 16 genes encoding for peroxidases were identified. Peroxidases are heme-containing enzymes that use hydrogen peroxide as the electron acceptor to catalyze a number of oxidative reactions (**Table 1**). Moreover, genes encoding for thioredoxins (H-type 7, H-type 8, and atypical ACHT5) and thioredoxin-dependent peroxidases were upregulated in *gsnor* plants (**Table 1**). The third subgroup of upregulated genes are encoding for 13 members of the Tau subfamily of GSTs (**Table 1**). The list of downregulated genes contains only a few transcripts related to redox-regulation, for example one putative peroxidase and a glutathione peroxidase 7 (**Table 1**). Moreover, four member of the Fe(II) and 2-oxoglutarate-dependent dioxygenase family with an oxidoreductase activity was found to be downregulated. In sum, the transcript profile analysis of *gsnor* plants suggests a pre-induced antioxidant system under normal growth condition, which can help to defend plants against subsequent oxidative stress.

**Table 1:** Candidate genes involved in H_2_O_2_ metabolism.

Gene	Fold-change	Protein
**Upregulated**		
AT4G16270	13.66	Peroxidase 40 (PER40) (P40)
AT1G44970	12.88	Peroxidase, putative
AT5G47000	11.94	Peroxidase, putative
AT5G64100	5.31	Peroxidase, putative
AT1G14540	5.27	Anionic peroxidase, putative
AT5G64110	5.15	Peroxidase, putative
AT5G64120	4.13	Peroxidase, putative
AT5G39580	3.84	Peroxidase, putative
AT3G03670	3.80	Peroxidase, putative
AT5G05340	3.60	Peroxidase, putative
AT4G33420	2.95	Peroxidase, putative
AT5G06720	2.92	Peroxidase, putative
AT4G08770	2.23	Peroxidase, putative
AT4G37520	2.12	Peroxidase 50 (PER50) (P50) (PRXR2)
AT5G19890	2.09	Peroxidase, putative
AT5G15180	2.05	Peroxidase, putative
AT1G60740	18.77	Peroxiredoxin type 2, putative
AT1G65970	6.99	TPX2 (thioredoxin-dependent peroxidase 2)
AT1G69880	6.20	ATH8 (thioredoxin H-type 8)
AT5G61440	3.31	ACHT5 (ATYPICAL CYS HIS RICH THIOREDOXIN 5)
AT1G59730	2.48	ATH7 (thioredoxin H-type 7)
AT2G29490	33.42	ATGSTU1 (GLUTATHIONE S-TRANSFERASE TAU 1)
AT1G17180	32.23	ATGSTU25 (GLUTATHIONE S-TRANSFERASE TAU 25)
AT2G29480	28.71	ATGSTU2 (GLUTATHIONE S-TRANSFERASE TAU 2)
AT1G17170	11.78	ATGSTU24 (GLUTATHIONE S-TRANSFERASE TAU 24)
AT2G29470	5.73	ATGSTU3 (GLUTATHIONE S-TRANSFERASE TAU 3)
AT1G69930	5.61	ATGSTU11 (GLUTATHIONE S-TRANSFERASE TAU 11)
AT1G59670	4.15	ATGSTU15 (GLUTATHIONE S-TRANSFERASE TAU 15)
AT3G09270	3.94	ATGSTU8 (GLUTATHIONE S-TRANSFERASE TAU 8)
AT1G74590	3.74	ATGSTU10 (GLUTATHIONE S-TRANSFERASE TAU 10)
AT2G29440	3.65	ATGSTU6 (GLUTATHIONE S-TRANSFERASE TAU 6)
AT2G29420	3.41	ATGSTU7 (GLUTATHIONE S-TRANSFERASE TAU 7)
AT1G69920	2.38	ATGSTU12 (GLUTATHIONE S-TRANSFERASE TAU 12)
AT2G29460	2.31	ATGSTU4 (GLUTATHIONE S-TRANSFERASE TAU 4)
**Downregulated**		
AT4G11290	-2.04	Peroxidase, putative
AT4G31870	-2.24	ATGPX7 (glutathione peroxidase 7)
AT4G23340	-5.61	Oxidoreductase, 2OG-Fe(II) oxygenase family protein
AT1G55290	-3.28	Oxidoreductase, 2OG-Fe(II) oxygenase family protein
AT1G49390	-2.59	Oxidoreductase, 2OG-Fe(II) oxygenase family protein
AT2G44800	-2.36	Oxidoreductase, 2OG-Fe(II) oxygenase family protein

*Gene expression analysis of redox-regulated genes by transcript profiling of *gsnor* under normal growing condition. Fold change (*p* < 0.05) represents transcript abundance in *gsnor* relative to WT plant.*

In line with the transcriptional data, we have analyzed the O_2_^-^ and H_2_O_2_ level in WT and *gsnor* plants after paraquat treatment. Ten days old seedlings grown on MS media were treated with 25 μM paraquat and vacuum infiltrated with either nitroblue tetrazolium (NBT) for O_2_^-^ detection (Doke, 1983) or DAB for H_2_O_2_ accumulation (Thordal-Christensen et al., 1997) (Supplementary Figure S3). No obvious difference in O_2_^-^ accumulation could be observed in paraquat-treated WT and *gsnor* plants (Supplementary Figure S3A). In contrast, H_2_O_2_ accumulation was lower in leaves of *gsnor* plants in comparison to WT plants (Supplementary Figure S3B) suggesting a higher capacity to metabolize H_2_O_2_ in *gsnor* line.

### H_2_O_2_-Dependent Inhibition of GSNOR Activity *In vitro*

**FIGURE 4:**
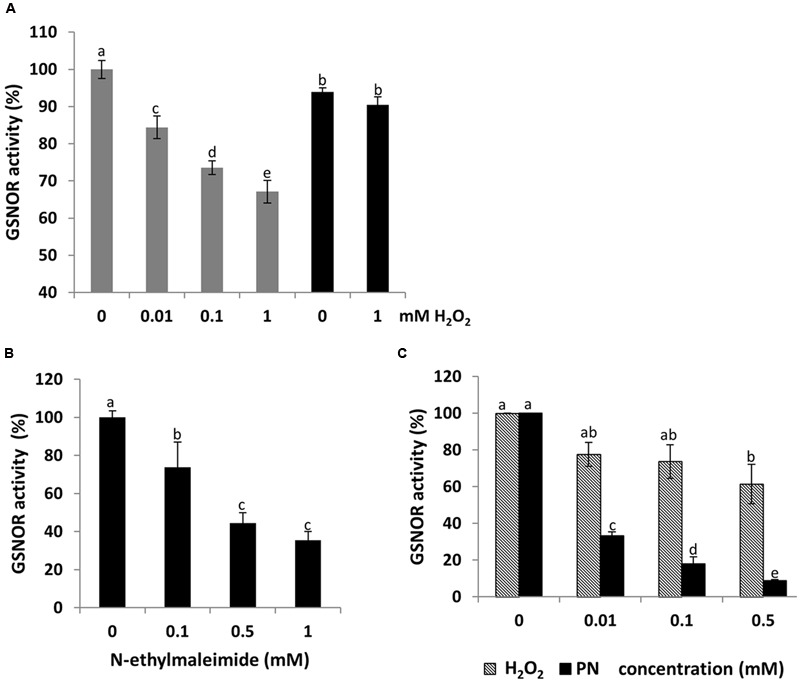
**Dose-dependent inhibition of GSNOR activity by oxidation *in vitro*.** Measurements of GSNO reducing activity of GSNOR protein. Recombinant GSNOR (1 μg) was incubated with indicated concentrations of H_2_O_2_ **(A)**, N-ethylmaleimide **(B)**, and peroxynitrite (PN) and H_2_O_2_ **(C)**. For restoring GSNOR activity, 10 mM DTT was added to the H_2_O_2_ inhibited enzyme **(A)**. Values are expressed as percentage of the control activity (at 0 mM: 87.5–110.9 μmol NADH min^-1^ mg^-1^ varied among independent purifications) and represent the mean ± SD calculated from three independent assays. Different letters indicate significant differences (*p* < 0.05, Anova).

To analyze whether H_2_O_2_ affect *Arabidopsis* GSNOR activity, recombinant protein was incubated with increasing concentrations of H_2_O_2_ (0.01, 0.1, and 1 mM). A dose-dependent reduction of the GSNOR activity was observed (**Figure 4A**). Treatment with 10 μM H_2_O_2_ caused already 15% inhibition, which further decreased to 35% in the presence of 1 mM H_2_O_2_. Incubation of inhibited GSNOR with 10 mM of the reducing agent DTT partly restored the activity, concluding that enzyme inhibition was a result of reversible and irreversible modifications of one or several redox-sensitive amino acid residues. Treatment with the sulfhydryl-blocking agent *N*-ethylmaleimide also inhibited the activity of GSNOR to 40% demonstrating that cysteine residues are important for its activity (**Figure 4B**). Peroxynitrite (ONOO^-^) is formed from the reaction of superoxide (O_2_^-^) and NO and acts as a potent oxidant on cysteine residues and as a nitrating agent on tyrosine residues of proteins (Arasimowicz-Jelonek and Floryszak-Wieczorek, 2011). Treatment of GSNOR with 10 μM of peroxynitrite reduced the activity by 70% (**Figure 4C**). Western blot analysis using 3-nitrotyrosine specific antibodies to detect tyrosine nitration of peroxynitrite-treated GSNOR protein did not show any nitration (data not shown) indicates that the peroxynitrite-dependent loss of GSNOR activity is probably due to the oxidation of cysteine(s) (Zeida et al., 2013).

### Structural Analysis of H_2_O_2_-treated GSNOR

**FIGURE 5:**
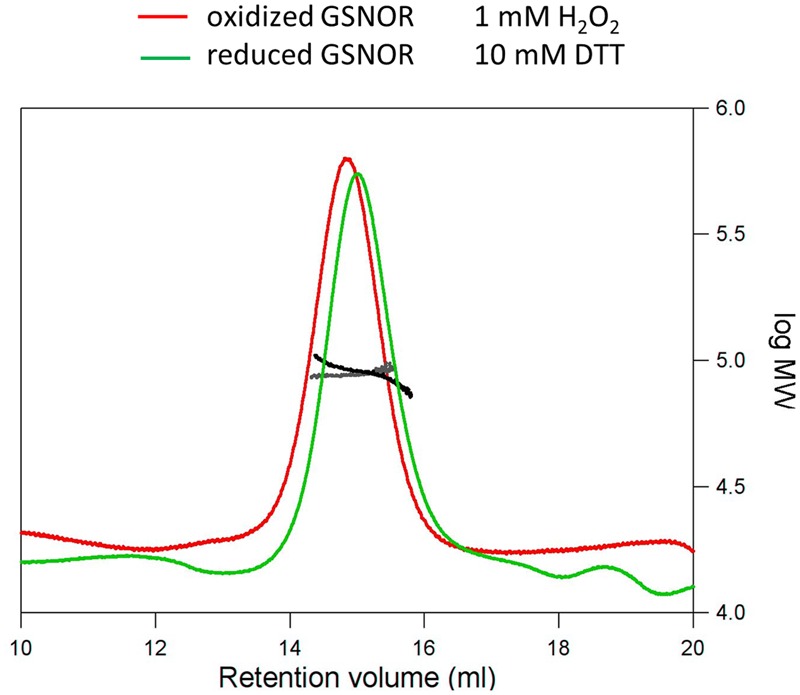
**Structural analysis of H_2_O_2_-treated GSNOR.** Determination of the molecular weight of reduced (green) and oxidized (red) GSNOR using size exclusion chromatography in combination with static light scattering. The refractive index and right angle light scattering signals were monitored and used to determine the molecular weight of the reduced (black) and oxidized (gray) protein. 100 μl of the protein samples were applied to a Superdex 200 10/300 GL column. Reduced (10 mM DTT) and oxidized (1 mM H_2_O_2_) GSNOR were analyzed at approximately 1.5 mg/ml and both eluted as a homodimer of 90.1 and 88.4 kDa, respectively (calculated Mr is 86 kDa).

As known from the crystal structure, GSNOR is a homodimer protein. To analyze, whether oxidative conditions cause changes in the native structure of GSNOR SLS experiments with reduced and oxidized recombinant GSNOR were performed. **Figure 5** shows that GSNOR protein is detected as dimer under both reducing (10 mM DTT) and oxidizing (1 mM H_2_O_2_) conditions with no significant difference between both states. The determined molecular weight was 90.1 and 88.4 kDa for reduced and oxidized GSNOR, respectively, which corresponds to the calculated weight of dimer (86 kDa for His_6_-GSNOR).

Shifts in the electrophoretic mobility of proteins are diagnostic for the presence of oxidative modifications, like cysteine oxidations or formation of disulfide bridges (Benezra, 1994; Mahoney et al., 1996; Despres et al., 2003). Therefore, we treated recombinant GSNOR with 10–500 μM H_2_O_2_ and DTT and investigated its running behavior by non-reducing SDS PAGE (Supplementary Figure S4). We could not observe a defined shift in the mobility of the oxidized proteins, but the more diffuse protein bands with increasing concentration of H_2_O_2_ may indicate the presence of several different oxidative modifications. Subsequent treatment of oxidized GSNOR with 10 mM DTT resulted in a sharp single band demonstrating the reversibility of the oxidative modifications.

### H_2_O_2_ Treatment of GSNOR Results in Oxidative Modification of Multiple Cysteine Residues

**FIGURE 6:**
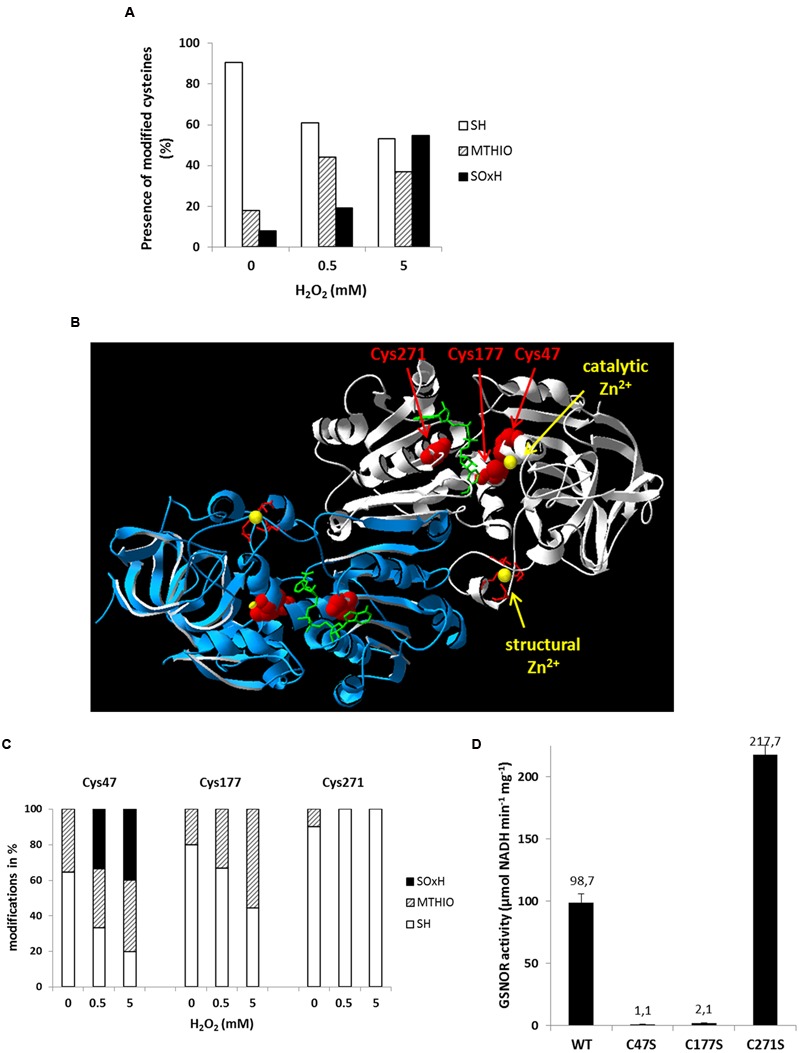
**Oxidative modifications of multiple cysteine residues of GSNOR correlate to reduced activity. (A)** nano LC-MS/MS analysis of cysteine residues of recombinant GSNOR oxidized with 0.5 and 5 mM H_2_O_2_. The portion of different modification is represented as the mean percentage of all detected peptides (modifications of individual cysteines are shown in Supplementary Figure S1). SH represents free cysteines, MTHIO-labeling shows reversibly modifications, and SOxH represents irreversibly oxidative modifications. **(B)** Percentage distribution of different modifications (SH, MTHIO, and SOxH) of Cys47, Cys177, and Cys271 residues of oxidized GSNOR by nano LC-MS/MS. **(C)** 3D structure of *Arabidopsis* GSNOR (PDB code: 3UKO) as a homodimer (two subunit is labeled by white and blue). Cysteine residues in the substrate-binding site are highlighted in red. The bound NAD^+^ cofactor is shown by green sticks. The catalytic and structural Zn^2+^ is labeled by yellow. **(D)** Specific enzyme activity was determined of WT GSNOR and cysteine mutants GSNOR^C47S^, GSNOR^C177S^, and GSNOR^C271S^. The mean values with SD of three determinations are presented in the graph.

The thiol group of cysteine residues is susceptible to oxidation resulting in a formation of sulfenic, sulfinic, or sulfonic acids, the latter two are irreversible modifications. Moreover, disulfide bridges can be formed. Since GSNOR has 15 cysteine residues, we analyzed their oxidative modifications by nano LC-MS/MS spectrometry (Supplementary Figure S5). Recombinant GSNOR was purified under reducing condition, than was oxidized with 500 μM H_2_O_2_ (equivalent to 100:1 molar ratio of H_2_O_2_ to GSNOR protein) and with 5 mM H_2_O_2_. Afterward, free cysteine residues were blocked with iodoacetamide and reversibly modified cysteine residues were reduced with DTT and labeled with *S*-methyl-methanethiosulfonate (MTHIO). After chymotryptic digestion, the cysteine containing peptides were analyzed for their modifications by nano-LC-MS/MS. Peptides containing Cys94, Cys99, Cys102, and Cys105 could not be detected at all. All other cysteine residues could be identified in its reduced form and the average amount of free thiols (-SH) was 90% in the water treated samples (**Figure 6A**). Increasing concentrations of H_2_O_2_ increased the amount of oxidized cysteine residues. We identified both reversibly (MTHIO-labeled) and irreversibly (sulfinic or sulfonic acids, SO_x_H) modified cysteine residues (Supplementary Figure S5). According to the increasing concentration of H_2_O_2_ the average frequency of MTHIO-labeled peptides increased from 17% to around 40% and the SO_x_H-modified cysteines from 8 to 55% (**Figure 6A**). Three cysteine residues (Cys47, Cys177, and Cys271) are located in the substrate-binding site of GSNOR highlighted in the three-dimensional structure of *Arabidopsis* GSNOR (**Figure 6B**). Cys47 and Cys177 are coordinating the catalytic Zn^2+^ together with His69 and water molecule. H_2_O_2_ treatment increased the amount of oxidative modifications for both Zn^2+^-coordinating cysteines (**Figure 6C**). Around 35% of Cys47 was already oxidized in water-treated GSNOR and the abundance of both MTHIO and SO_x_H-modification increased significantly in the presence of 0.5 and 5 mM H_2_O_2_ (66 and 80%, respectively). This indicates that Cys47 is very sensitive for oxidation. The other Zn^2+^-coordinating cysteine Cys177 also showed increased reversible oxidation up to 55% after 5 mM H_2_O_2_ treatment. Interestingly, Cys271, which is located in the NAD^+^ cofactor-binding site, was mainly found in its reduced form independent of the treatment. To analyze the importance of these three cysteines, we have generated GSNOR mutants, where these cysteine residues were exchanged by serine resulting GSNOR^C47S^, GSNOR^C177S^, and GSNOR^C271S^. GSNOR^C47S^ and GSNOR^C177S^ showed drastically reduced specific activity (100-fold less and 50-fold less, respectively) compared to WT GSNOR (**Figure 6D**) demonstrating the importance of these two cysteines. In contrast, the mutation of Cys271 resulted in twofold increase of the specific activity.

### H_2_O_2_-Induced Zn^2+^ Release of GSNOR

**FIGURE 7:**
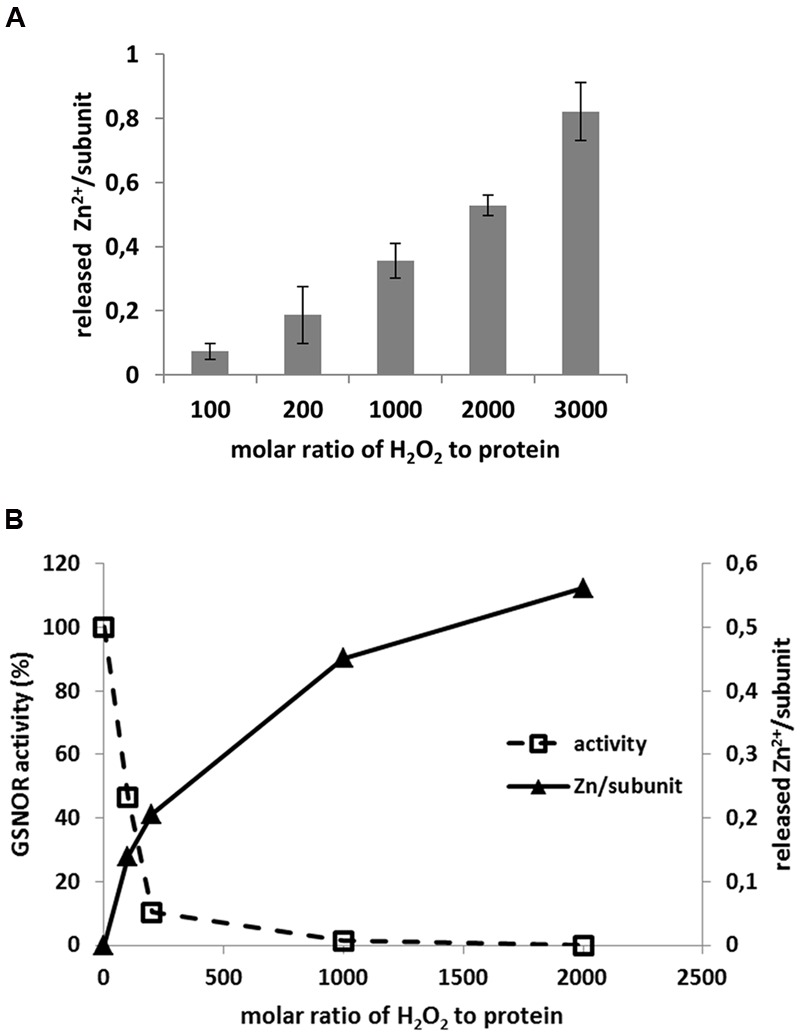
**H_2_O_2_-induced Zn^2+^ release of GSNOR protein. (A)** Determination of Zn^2+^ release of recombinant GSNOR (50–100 μg) incubated for 1 h with increasing molar excess of H_2_O_2_ as indicated. The measured Zn^2+^ was first corrected by untreated GSNOR sample (background correction) and then calculated to one subunit of GSNOR. **(B)** Representative experiment for parallel measurement of GSNOR activity and Zn^2+^ release using the same oxidized sample as indicated. GSNOR activity is presented as percentage of untreated protein. The released Zn^2+^ is calculated to one subunit of GSNOR.

*S*-nitrosoglutathione-reductase contains two Zn^2+^ per subunit; one is located in the active center of protein called catalytic Zn^2+^, the other one is structural Zn^2+^. The catalytic Zn^2+^ is coordinated by Cys47 and Cys177 and both are sensitive for oxidation (**Figure 6C**). Therefore, we tested whether oxidative inhibition of enzyme correlates with Zn^2+^-release. Treatment of recombinant GSNOR protein with H_2_O_2_ resulted in Zn^2+^-release in a H_2_O_2_ concentration-dependent manner (**Figure 7A**). We observed a maximum release of 0.82 Zn^2+^ (±0.16) calculated for one subunit of the dimer with the highest molar excess of H_2_O_2_ (3000-fold excess corresponded to 20 mM H_2_O_2_). The activity measurement of the H_2_O_2_-treated GSNOR showed that the enzyme was completely inhibited by 1000-fold molar excess of H_2_O_2_ (**Figure 7B**). This corresponds to a release of 0.4 Zn^2+^/subunit (**Figure 7B**) suggesting a correlation between activity loss and Zn^2+^-release. We could not measure higher Zn^2+^-release than 0.98 Zn^2+^/subunit indicating that the second Zn^2+^ atom (the structural one) is probably not affected by oxidation. Interestingly, excess of external Zn^2+^ prevented H_2_O_2_-caused inhibition of GSNOR activity (Supplementary Figure S6), further confirming, that loss of Zn^2+^ results in loss of GSNOR activity.

## Discussion

The term oxidative stress describes the temporary imbalance of the cellular redox homeostasis due to enhanced accumulation of ROS triggering both signaling events and damaging processes (Foyer and Noctor, 2013). A change in ROS homeostasis and the associated shift in the redox state are induced primarily by external environmental influences during various abiotic and biotic stress treatments summarized in Mittler (2002). The major posttranslational protein modifications arising from interaction with ROS are oxidation of sulfur-containing residues (cysteine, methionine) and aromatic residues (tyrosine, tryptophan), carbonylation reactions and formation of disulfide bridges (Rinalducci et al., 2008; Waszczak et al., 2015). Besides ROS, reactive nitrogen species are very important signaling molecules in plants involved in biotic and abiotic stress responses. GSNOR activity controls intracellular levels of GSNO and *S*-nitrosylated proteins and the physiological importance of GSNOR in fine-tuning NO/SNO levels during many stress responses and in plant growth and development is well described. To investigate the effect of oxidative modifications on individual proteins is crucial in understanding the signaling responses under different stress conditions. We demonstrate that GSNOR activity is inhibited by H_2_O_2_ *in vitro* or paraquat *in vivo* (**Figures 1A** and **4A**), providing a new evidence for crosstalk between ROS and NO signaling. H_2_O_2_-dependent inhibition of GSNOR activity was also observed in *Baccaurea ramiflora* (Burmese grape) investigating chilling stress (Bai et al., 2012). Moreover, alcohol dehydrogenase 1 (YADH1) from *Saccharomyces cerevisiae* was also inhibited by H_2_O_2_ *in vitro* due to oxidative modifications of specific cysteine residues (Men and Wang, 2007). This enzyme is structurally related to GSNOR (class III alcohol dehydrogenase). After H_2_O_2_-treatment, Cys43 and Cys153 of YADH1 were oxidized and three disulfide bonds (Cys43–Cys153, Cys103–Cys111, Cys276–Cys277) were detected (Men and Wang, 2007). Cys43 and Cys153 of YADH1 correspond to *Arabidopsis* GSNOR Cys47 and Cys177 and they are conserved residues in class III alcohol dehydrogenases coordinating the catalytic Zn^2+^. In correlation with YADH1, we also observed oxidative modifications of these two residues (reversible and/or SO_2_H and SO_3_H) by MS analyses of H_2_O_2_-treated *Arabidopsis* GSNOR (**Figure 6**). Moreover, our results show that nearly all detected cysteine residues were accessible to oxidative modification in a dose-dependent manner (Supplementary Figure S5). In contrast to YADH1, we could not detect a disulphide formation of *Arabidopsis* GSNOR by a shift on non-reducing SDS-PAGE (Supplementary Figure S4) and also not by MS (data not shown). Substitution of Cys47 and Cys177 to serine residues resulted in a loss of GSNOR activity (**Figure 6D**) indicating the importance of a Zn^2+^-thiolate catalytic center. Early biochemical studies in mammals showed evidence that oxidation of cysteines of zinc-finger transcription factors can abolish DNA binding and transcriptional functions (Webster et al., 2001). Superoxide-induced Zn^2+^ release has also been demonstrated in the zinc finger motif of protein kinase C (Knapp and Klann, 2000). Furthermore, investigation of different oxidants on the oxidative Zn^2+^ release in YADH1 revealed an inverse correlation between alcohol dehydrogenase activity and the released Zn^2+^ (Daiber et al., 2002). The strongest oxidant was peroxynitrite leading to release of one zinc atom/subunit of YADH1, following H_2_O_2_ and the less effective was NO. Oxidation of recombinant *Arabidopsis* GSNOR by H_2_O_2_ also resulted in a Zn^2+^ release (**Figure 7**). Similarly, *Arabidopsis* GSNOR contains two Zn^2+^ per subunit, however, we observed the release of only one Zn^2+^/subunit at the highest excess of H_2_O_2_ (at molar excess of 3000). Since the Zn^2+^-release has been accompanied by loss of activity we assumed that most likely Zn^2+^ from the active center of the protein is released. Moreover, Cys47, which is involved in coordinating the catalytic Zn^2+^, is very sensitive to oxidation (**Figure 6C**). The second Zn^2+^ (structural Zn^2+^) is coordinated by four cysteine residues (Cys94, Cys99, Cys102, and Cys105) and is not involved in the enzymatic activity of GSNOR. Interestingly, *S*-nitrosation of conserved non-zinc coordinating cysteines (Cys10, Cys271, and Cys370) were reported very recently and this modification was shown to cause a catalytic inhibition of *Arabidopsis* GSNOR (Guerra et al., 2016).

To analyze the ROS-induced inhibition of *Arabidopsis* GSNOR *in vivo*, the bipyridium herbicide paraquat was used as a ROS-inducing agent (Vaughn and Duke, 1983). The redox cycling of paraquat with molecular oxygen produces superoxide radical, which is then mainly dismutated by superoxide dismutase (SOD) to H_2_O_2_ (Bus and Gibson, 1984). However, in the presence of NO, peroxynitrite is formed from the reaction between O_2_^-^ and NO, which is approximately six-times faster than the dismutation by SOD (Pacher et al., 2007). ONOO^-^ is a powerful oxidant and nitrosating compound in the cellular environment modifying amino acids, nucleic acids, low and high molecular weight thiols and phospholipids. Paraquat reversibly inhibits GSNOR activity (**Figure 1A**) resulting in enhanced levels of SNOs (**Figure 2A**). Plants that lack GSNOR activity are more tolerant toward paraquat than WT plants, which develop cell death phenotype germinated on 0.5–1 μM paraquat-containing media (**Figures 1C,D**) (Chen et al., 2009) suggesting an activated resistance mechanisms in *gsnor* plants. Enhanced levels of cellular SNOs in *gsnor* in comparison to WT plants (**Figure 2A**) might be responsible for the observed tolerance against paraquat-induced oxidative stress. In correlation, SNO levels increased more than twofold in WT plants after paraquat treatment (**Figure 2A**) providing evidence for ROS-induced inactivation of GSNOR *in vivo.* Co-treatment of NO donor sodium nitroprusside and paraquat during germination of WT plants resulted in increased resistance to paraquat supports this hypothesis (Chen et al., 2009). A protective effect of NO against paraquat-induced oxidative stress was also described in potato and rice after incubation with NO-releasing compounds, however, the exact mechanism is not provided (Beligni and Lamattina, 1999; Hung et al., 2002). Normally, higher SNO/NO levels in *gsnor* plants should increase the production of ONOO^-^ during paraquat treatment. In contrast, we observed a reduced tyrosine nitration level as a marker for ONOO^-^ production in the paraquat-treated *gsnor* plant (Supplementary Figure S1B). This result indicates that either the production or the turnover of ONOO^-^ is affected by excess NO/SNO. It was demonstrated in soybean cells that ONOO^-^ is not a determining factor of hypersensitive cell death, but the common action of NO and H_2_O_2_ (Delledonne et al., 2001). The paraquat-treated *gsnor* mutant accumulates lower amount of H_2_O_2_ than the WT plant (Supplementary Figure S3) supporting the scavenging function of NO to decrease excess level of ROS species. On one side, peroxynitrite formation can be a mechanism to consume superoxide thereby protecting biomolecules from oxidation and preventing further ROS production (Kanner et al., 1991; Wink et al., 2003). On the other side, ONOO^-^ can inhibit several isoforms of SOD (Holzmeister et al., 2015) resulting in less H_2_O_2_ production. Besides the scavenging function of NO, the plant cell can overcome elevated ROS levels by activating the antioxidant system. GSH is one of the major low molecular weight thiol, which reacts rapidly to changing stress situations and is crucial to maintain cellular redox balance. Both, loss of GSNOR function and fumigation with NO enhanced GSH level (**Figures 3A,C**) assuming that NO/SNO is able to stimulate the GSH biosynthesis pathway. These measurements coincide with previous reports demonstrating a higher amount of GSH in roots of *Medicago truncatula* (Innocenti et al., 2007) and maize leaves (Mello et al., 2012) after GSNO and SNP treatment, respectively. In both cases, an enhanced expression of the γ-glutamylcysteine synthetase and GSH synthetase gene was detectable suggesting a NO-dependent transcriptional regulation of GSH production. Together with a twofold higher glutathione content in *gsnor* plants, we measured increased glutathione reductase activity (**Figure 3B**), which is responsible for the recovery of GSH and thus the maintenance of the redox homeostasis. Assuming that the GR activity is involved in GSH regeneration, the *gsnor* plants would therefore be able to provide more reducing equivalents needed for the stress response (**Figure 3A**). Moreover, increased conjugase activity of GST was measured in *gsnor* plants with and without paraquat treatment (**Figure 3B**). Enhanced GST activity could be observed in response to different abiotic and biotic stimuli and their activity is important to protect plants against oxidative damage (Sappl et al., 2009). The induction of the antioxidant system in *gsnor* plants was further demonstrated by a transcript profile analysis. Comparison of WT and *gsnor* plants under normal growth condition revealed an enhanced expression of genes involved in antioxidant processes in *gsnor* plants (**Table 1**). The up-regulated genes of peroxidases or GSTs are markers for oxidative stress and/or H_2_O_2_ signaling (Vanderauwera et al., 2005; Queval et al., 2012). However, little is known about the exact physiological function of these enzymes during oxidative stress. Interestingly, several members of the Tau class GSTs are also upregulated during paraquat and H_2_O_2_ treatment of *Arabidopsis* seedlings (Genevestigator At-413 and Genevestigator At-185). Moreover, using a yeast two-hybrid approach a tomato cDNA library was screened for “proteins” protecting yeast from prooxidant–induced cell death. In this screen five homologous Tau class GSTs were identified concluding that especially this class of GST proteins has a protective function in oxidative stress response (Kilili et al., 2004). The fact that the expression of 13 members of the Tau subfamily of GSTs is upregulated in *gsnor* plants in comparison to WT plants suggests that these genes are regulated by SNO/NO and are important for protection against oxidative stress. Although peroxisomal catalases and the ascorbate-glutathione pathway play a primarily role in the metabolism of H_2_O_2_ (Mhamdi et al., 2010; Noctor et al., 2012), we did not observed any changes in the expression of catalases or genes related to the ascorbate-glutathione-dependent pathway like APX or dehydroascorbate reductases. However, several other classes of antioxidative peroxidases exist that can reduce H_2_O_2_ and/or organic peroxides. These include thioredoxin-, or glutathione-peroxidases, and glutathione-*S*-transferases (Dietz, 2003; Dixon et al., 2009). Based on our microarray data an alternative pathway involving SNO/NO-induced thioredoxin- and/or glutathione-dependent peroxidases might be present and result in activation of the antioxidative system.

Furthermore, a thiol protective role of *S*-nitrosylation has been reported in animals (Evangelista et al., 2013). Formation of higher order irreversible oxidative modifications, such as sulfinic and sulfonic acids were prevented by *S*-nitrosylation. Recent paper has provided evidence that *S*-nitrosylation of *Arabidopsis* APX1 enhances its activity to scavenge H_2_O_2_ and to increase resistance to oxidative stress (Yang et al., 2015). *S*-Nitrosylation of pea APX also enhanced its enzyme activity in saline stress (Begara-Morales et al., 2014). Moreover, the activity of NADPH oxidase is inhibited by *S*-nitrosylation, resulting in the reduction in ROS biosynthesis during immune responses (Yun et al., 2011). Interestingly, activity of *Arabidopsis* GSNOR is inhibited by *S*-nitrosylation demonstrating that SNOs control its own scavenging by modulating GSNOR activity (Frungillo et al., 2014; Guerra et al., 2016).

In sum, we demonstrated that GSNOR activity can be inhibited *in vitro* by H_2_O_2_, as well as *in vivo* by paraquat, which is accompanied by a significant change in NO homeostasis. The observed increase in cellular SNOs consequently leads to induction of NO-dependent signaling mechanisms, resulting in GSH accumulation, enhanced activity of GSH-related enzymes and finally in a protection against oxidative stress. All these findings substantiate the physiological importance of GSNOR in fine-tuning the levels of NO/SNO during plant growth and development and also in many stress response reactions.

## Author Contributions

IK, CH, and CL designed research. IK, CH, MW, AG, GR, TF, GK, and EL performed research. IK, CH, MW, AG, TF, RH, GA, JD, and CL analyzed data. IK, CH, and CL wrote the paper.

## Conflict of Interest Statement

The authors declare that the research was conducted in the absence of any commercial or financial relationships that could be construed as a potential conflict of interest.

## Footnotes

1 http://www.ebi.ac.uk/arrayexpress
