# Supplementary material for: ROS-Mediated Inhibition of S-nitrosoglutathione Reductase Contributes to the Activation of Anti-oxidative Mechanisms
Source: Front Plant Sci. 2016 Nov 10;7:1669. doi: 10.3389/fpls.2016.01669 (PMC5102900; doi:10.3389/fpls.2016.01669)
Supplement: Supplementary file 3 [file Image_1.pdf]

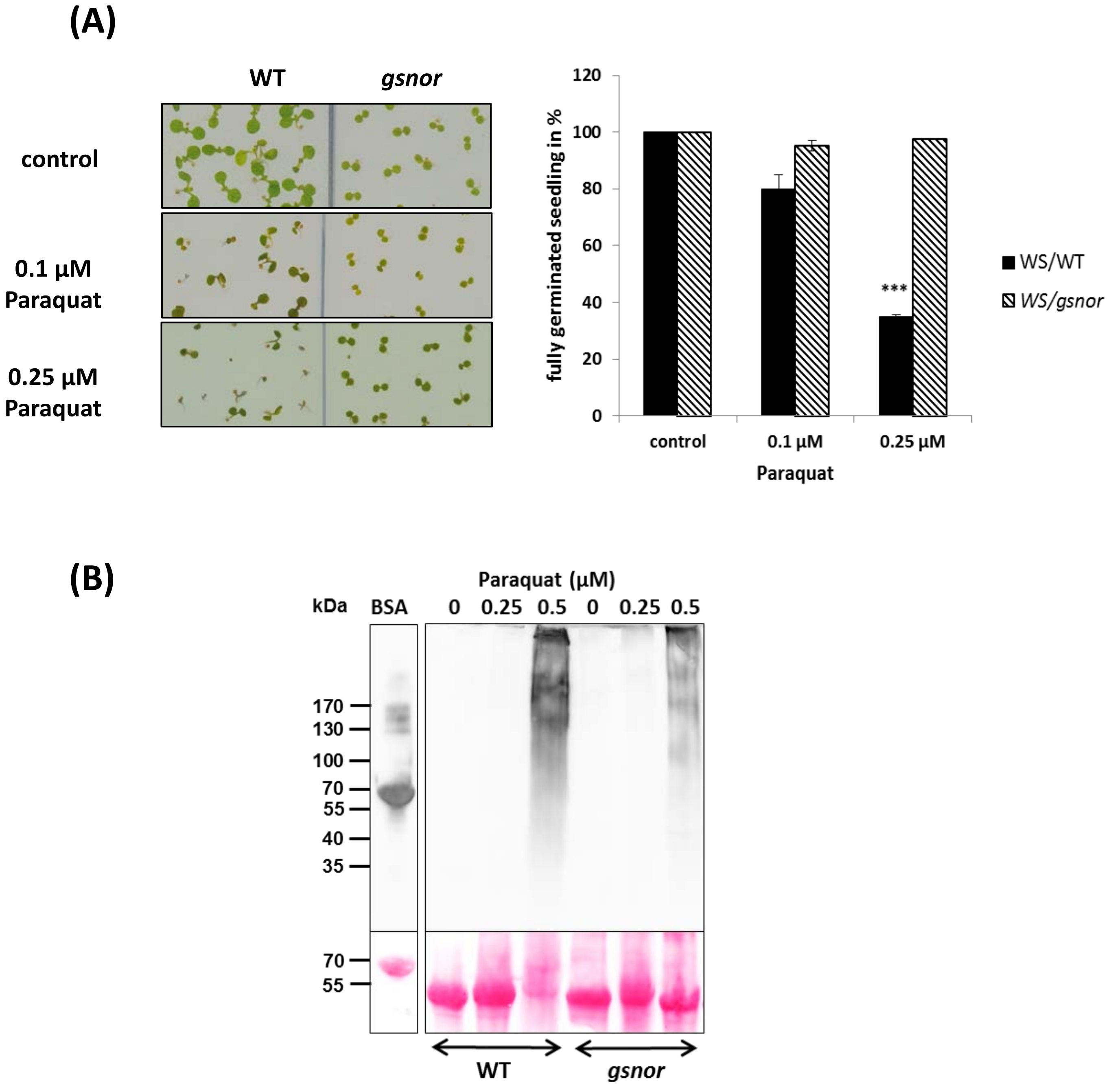

Figure S1: (A) Germination rates of one-week-old Wassilewskija (WS) WT and *gsnor* mutant plants grown on 0, 0.1 µM, and 0.25 µM paraquat-containing media. The germination rate was calculated by counting fully germinated seedlings with open cotyledons and is presented in percentage of the control (without paraquat). *** ($p<0.001$) indicate significant differences between WT and *gsnor*. **(B)** Western blot using anti-nitrotyrosine antibody to detect peroxynitrite production. Total protein extract was prepared from WT and gsnor seedlings grown on control and paraquat-containing media (0.25 µM and 0.5 µM) for two weeks. Nitrosated BSA was used as positive control (first lane, BSA). Molecular weight marker is indicated at the left side. Ponceau staining of Rubisco protein is shown on the bottom panel.

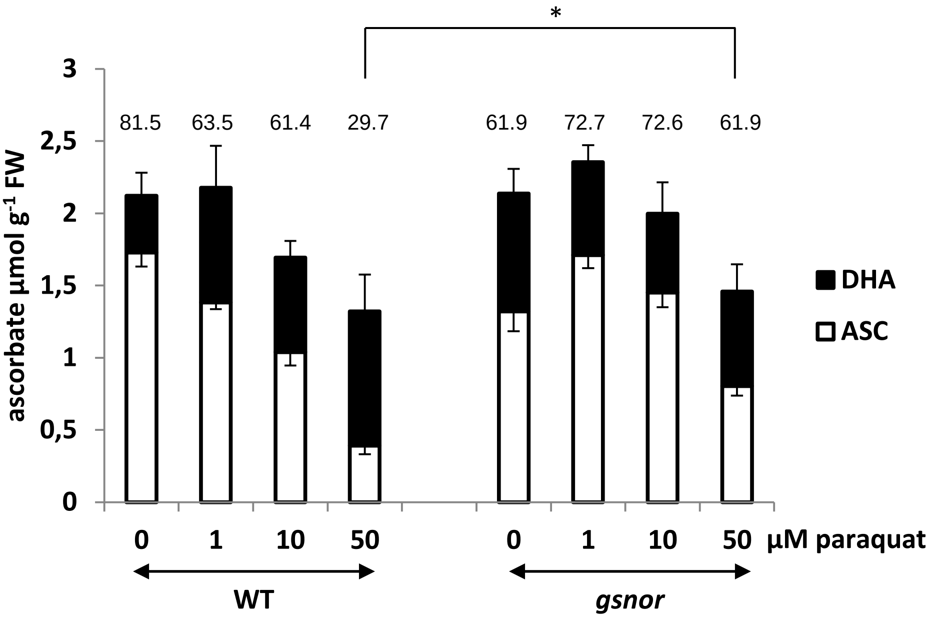

Figure S2: Determination of reduced (ascorbate, ASC, white bars) and oxidized ascorbate (dehydroascorbate, DHA, black bars) in WT an *gsnor* mutant after paraquat treatment. 4-week-old Arabidopsis plants were sprayed with water (0-control), 1 µM, 10 µM and 50 µM paraquat for 24 h (A). Values were normalized against fresh weight (FW) and represent the mean ± SE calculated from five biological replicates. Numbers above each bar indicate percentage reduction states of ascorbate (%). * ($p<0.05$) represent significant difference between WT and *gsnor* sample treated with 50 µM paraquat.

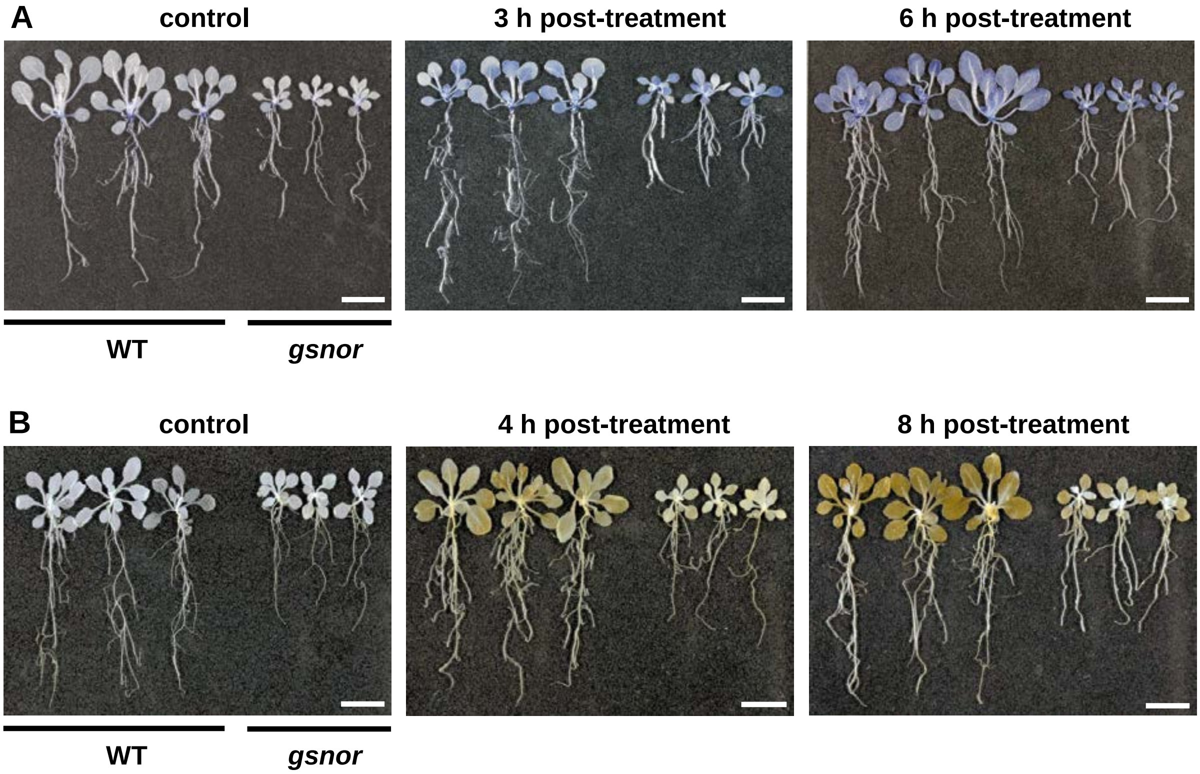

Figure S3: Histochemical detection of superoxide and hydrogen peroxide after paraquat treatment. 10 days old seedlings from WT plants and *gsnor* mutants cultured under sterile conditions on MS media were sprayed with 25 µM paraquat and vacuum infiltrated with either NBT staining solution (**A**, detection of O_2^-) or DAB staining solution (**B**, detection of H_2O_2) at the different times indicated, while control treatments were carried out with water instead of paraquat. To visualize blue formazan deposits and brown polymerisates, seedlings were incubated in boiling ethanol to get rid of chlorophyll pigments (bar=1 cm).

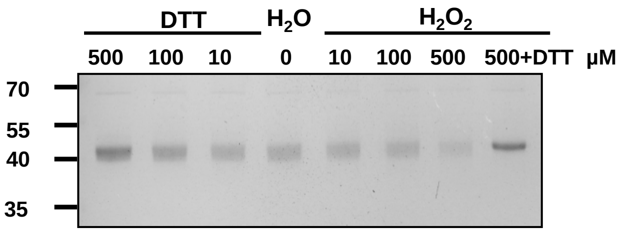

Figure S4: Running behavior of oxidized and reduced GSNOR protein by non-reducing SDS-PAGE. 0.5 µg of recombinant GSNOR protein was incubated with different concentrations of DTT and H_2O_2 as indicated for 1 hour. To demonstrate the reversibility of the oxidation, the sample oxidized with 500 µM H_2O_2 was reduced with 5 mM DTT and then separated on 12% non-reducing SDS-PAGE. The relative masses of protein standards are shown on the left.

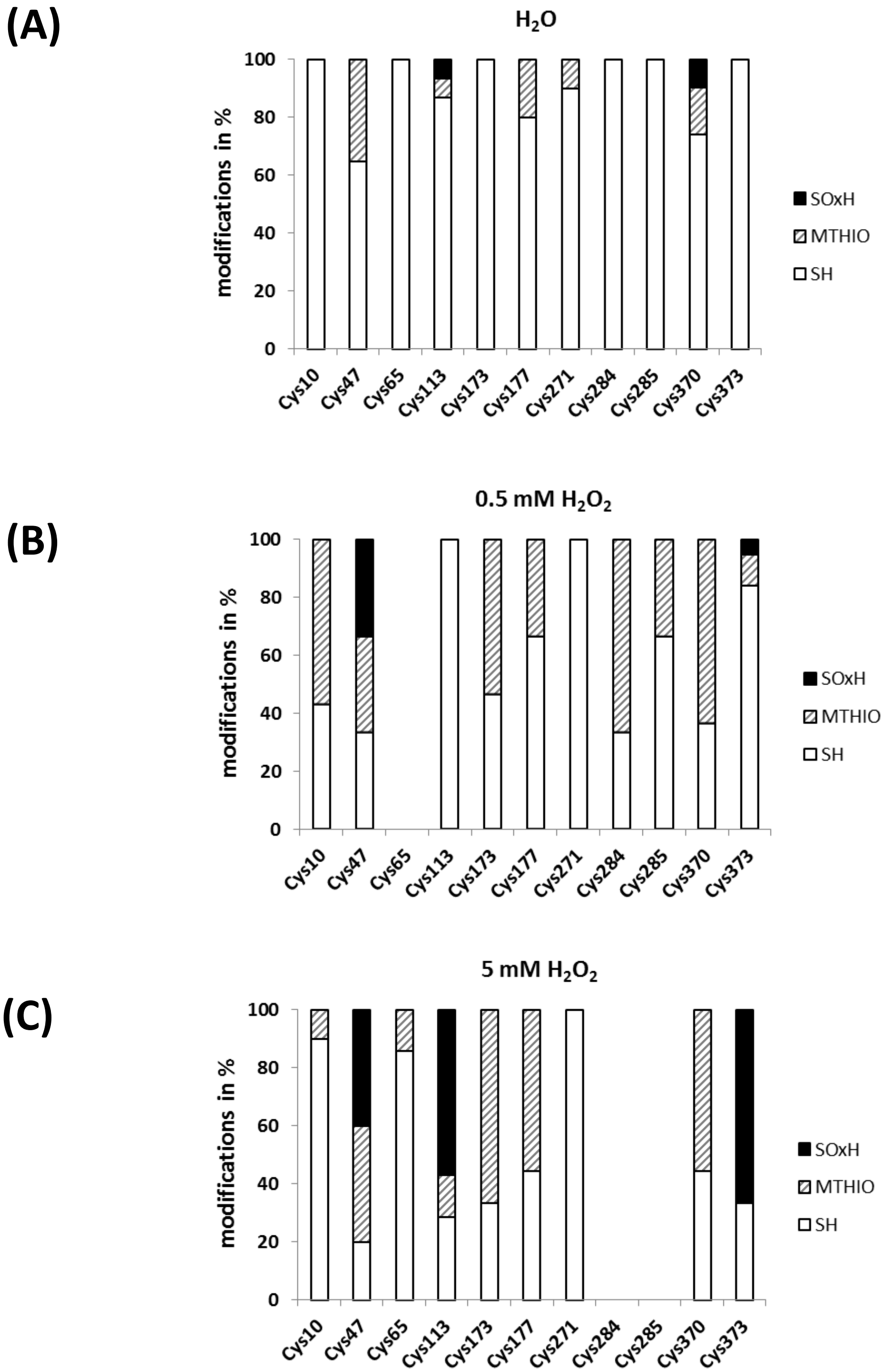

Figure S5: Nano LC-MS/MS analyses of cysteine residues of recombinant GSNOR treated with 0 mM (A), 0.5 mM (B) and 5 mM H_2O_2 (C). The portion of the different modifications is represented as the mean percentage of all detected peptides. SH represents free cysteines, MTHIO-labeling shows reversibly modifications, and SOxH represents irreversibly oxidative modifications.

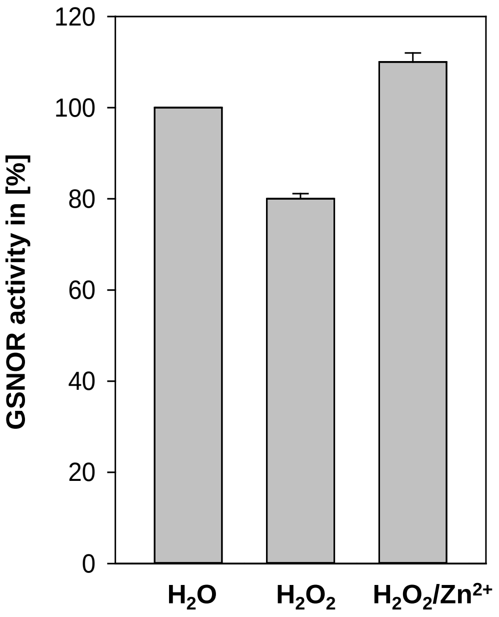

Figure S6: Effect of H_2O_2 on GSNOR activity in presence of excess Zn^{2+}. GSNOR was treated with 0,5 mM H_2O_2 in presence of 0,5 µM $ZnSO_4$ for 20 min. Excess H_2O_2 and $ZnSO_4$ was remove using ZebaSpin columns and GSNOR activity was determined. The activity of water treated GSNOR (control) was set to 100%. Values represent the mean of three measurements.
